# Solitude can be good—If you see it as such: Reappraisal helps lonely people experience solitude more positively

**DOI:** 10.1111/jopy.12887

**Published:** 2023-09-19

**Authors:** Micaela Rodriguez, Samuel Pratt, Benjamin W. Bellet, Richard J. McNally

**Affiliations:** ^1^ Department of Psychology University of Michigan Ann Arbor Michigan USA; ^2^ Department of Psychology Harvard University Cambridge Massachusetts USA

**Keywords:** loneliness, isolation, solitude, emotion, mindset, reappraisal, emotion regulation

## Abstract

**Objective:**

Solitude is a common experience that can elicit both positive (e.g., relaxation) and negative (e.g., loneliness) emotions. But can changing the way we think about solitude improve its emotional effects? In a previous study, our team found that positively reframing solitude buffers against a reduction in positive affect when alone. Yet, it is unknown whether people who are lonely—and thus more likely to experience solitude negatively—benefit from modifying their beliefs about being alone.

**Method:**

Here, we test whether reframing solitude as a beneficial experience or de‐stigmatizing loneliness helps people experiencing moderate‐to‐severe loneliness (*N* = 224) feel more positive emotion and less negative emotion during solitude. We randomly assigned participants to read about either the benefits of solitude, the high prevalence of loneliness, or a control topic. Then, participants spent 10 min alone in the laboratory. State affect was assessed before and after the solitude period.

**Results:**

Across conditions, the solitude period reduced high‐arousal positive (e.g., excited) and high‐arousal negative (e.g., anxious) affect. Notably, people who read about the benefits of solitude experienced a significantly larger increase in low‐arousal positive affect compared with the control condition.

**Conclusion:**

Our findings indicate that lonely individuals can more readily reap the emotional benefits of solitude when they reframe solitude as an experience that can enhance their well‐being.

## 1 INTRODUCTION

Solitude is a historically elusive phenomenon; the Buddha famously viewed solitude as a prerequisite for inner peace, yet others warn of a nationwide “loneliness epidemic” that results from spending too much time alone (Brooks, 2018). Although there is no consensual definition in the literature (Weinstein et al., 2022), we conceptualize solitude as the state of being physically alone, separated from others (Coplan et al., 2021). Under this definition, solitude is a ubiquitous phenomenon—across ages, cultures, and contexts, humans experience time alone for a myriad of reasons. But how does solitude affect our well‐being?

Consistent with diverging conceptualizations of solitude, psychological research indicates that solitude can be both an asset and a liability to our health and well‐being. For instance, solitude can increase life satisfaction (Long et al., 2003), promote emotion regulation (Nguyen et al., 2018; Rodriguez et al., 2020), and reduce stress (Larson & Lee, 1996). However, solitude may also lead to negative affect (Lay et al., 2019), boredom (Wilson et al., 2014), anxiety (Rubin et al., 2002), and loneliness (Williams & Nida, 2011). The psychological effects of solitude are shaped by diverse factors, including individual differences (e.g., introversion), self‐perceptions (e.g., social self‐efficacy), demographic characteristics (e.g., age), and sociocultural context (e.g., social norms; Coplan et al., 2021; Lay et al., 2019; Rodriguez et al., 2022; Weinstein et al., 2021).

An often‐overlooked factor that may influence whether solitude is experienced positively or negatively is how people *think* about solitude (Rodriguez et al., 2020). Indeed, decades of evidence indicate that our beliefs shape subjective experiences and health outcomes (Ellis, 1991). For instance, mindset research suggests that success is not only a function of intelligence and ability, but also of our beliefs about the malleability of our own abilities (Dweck, 2016; Yeager et al., 2019). In addition, placebo research demonstrates that simply believing that a sugar pill has therapeutic properties can powerfully reduce physical pain and psychological distress (Stoessl & de la Fuente‐Fernández, 2004). In this vein, it is possible that how we think about solitude shapes its emotional effects.

Although solitude is not inherently harmful, it tends to be viewed negatively in American culture. Empirical evidence indicates that solitary individuals are regarded as inferior to, and less worthy than, individuals who are more sociable (Kerr & Stanley, 2021; Lau & Gruen, 1992). Furthermore, extraversion is highly prized in American society (van Zyl et al., 2018); according to cross‐cultural research, extraversion robustly predicts life satisfaction among Americans but is unrelated to life satisfaction in non‐North American samples (e.g., Germany, Japan, United Kingdom; Kim et al., 2018). Moreover, even brief periods of solitude are uncomfortable and emotionally distressing for many Americans (Wilson et al., 2014). In the words of philosopher Philip Koch (1994, p. 220), American society views being alone as “unnatural, pathological, and dangerous.” And yet, the capacity to be alone is an important skill if one wishes to reap the cognitive and affective benefits that solitude can offer (Winnicott, 1958). Further, no matter how assiduously we may try to avoid it, spending time alone is virtually inevitable in the modern world.

Such negative perceptions of being alone may prevent individuals from experiencing the well‐documented benefits of solitude (e.g., relaxation). Fortunately, ample research suggests that our beliefs are malleable and that changing how we interpret situations in our lives (e.g., via cognitive reappraisal) can powerfully shape emotion, health, and performance (McRae & Gross, 2020). For instance, reframing pre‐test anxiety as beneficial to performance significantly improves individuals' scores on the Graduate Record Examination (GRE; Jamieson et al., 2013). As prior research has conceptualized the capacity for positive solitude as a skill that can be developed (Palgi et al., 2021), there may be great potential for cognitive change to improve the way that individuals think about, and consequently experience, solitude.

Considering this possibility, our team previously conducted an experiment to examine whether reframing solitude as beneficial to one's well‐being helps people experience a period of solitude more positively (Rodriguez et al., 2020). We randomly assigned individuals to a control condition or either of two reappraisal conditions which aimed to help people reframe solitude. We found that, across conditions, people experienced a decrease in positive affect after spending 10 min alone. Notably, however, individuals who read about the benefits of solitude experienced a *smaller decrease* in positive affect than controls. In other words, reframing time alone as a positive experience buffered people against the negative emotional effects of isolation.

These results provide preliminary evidence that thinking about solitude in a positive way improves how people experience their time alone (Rodriguez et al., 2020). However, it remains unknown whether these results generalize to other populations, such as individuals experiencing high levels of loneliness. Do the benefits of cognitively reframing solitude apply to people who are lonely, and who may therefore view solitude as inherently distressing, pathogenic, and a reminder of their social inadequacy (Burger, 1995)? The present study aims to address this question.

## 2 PRESENT STUDY

Here, we build on our team's prior work (Rodriguez et al., 2020) by testing whether brief reappraisal interventions—which aim to help people modify how they think about solitude—allow lonely individuals to experience a brief period of solitude more positively. As prior work demonstrates that solitude is most strongly associated with low‐arousal affect—both positive (e.g., *relaxed*) and negative (e.g., *bored*) in valence—and weakly associated with high‐arousal affect (e.g., *joyful*, *afraid*), we are primarily interested in the effects of time alone on low‐arousal states (Birditt et al., 2019).

We also tested the potential moderating effects of trait reappraisal, perceptions of inferiority, and compulsive social media use on changes in affect. Prior research indicates that these individual differences are robustly associated with loneliness and may shape experiences of solitude (O'Day & Heimberg, 2021; Preece et al., 2021). For instance, individuals who do not regularly use cognitive reappraisal in daily life (i.e., are low in trait reappraisal) generally feel lonelier and may benefit most from a reappraisal intervention (Preece et al., 2021; Rodriguez et al., 2020). In addition, people who perceive themselves as inferior to others may not enjoy time with themselves and may be more likely to experience solitude negatively (Long et al., 2003; Yang et al., 2020). Finally, people who feel lonely may be more likely to use social media to cope with the pain of isolation (Cauberghe et al., 2021); however, excessive social media use is associated with an increase in loneliness over time (Kross et al., 2013; Marttila et al., 2021).

Our primary research questions and hypotheses are as follows:

*RQ 1*. Does reading a passage about the benefits of solitude or the high prevalence of loneliness change the emotional effects of a brief period of solitude among lonely people?

Learning about the benefits of solitude (*Solitude Benefits condition*) or the high prevalence of loneliness (*Loneliness De‐Biasing condition*) will lead lonely people to experience a larger increase in low‐arousal positive affect and a larger decrease in low‐arousal negative affect (compared to the *Control condition; reading a passage unrelated to time alone*) after 10 min of solitude.

*RQ 2*. Do theoretically relevant individual differences—trait cognitive reappraisal, perceptions of inferiority on social media, and compulsive social media use—moderate the relationships between the experimental conditions and changes in low‐arousal positive and negative affect?

In the experimental conditions, individuals who use cognitive reappraisal *less* frequently (i.e., are low in trait reappraisal) will experience larger increases in low‐arousal positive affect and larger decreases in low‐arousal negative affect after spending 10 min alone (compared to the Control condition).

In the experimental conditions, individuals who perceive themselves as *more* inferior to others on social media will experience larger increases in low‐arousal positive affect and larger decreases in low‐arousal negative affect after spending 10 min alone (compared to the Control condition).

In the experimental conditions, individuals who report *greater* compulsive social media use will experience larger increases in low‐arousal positive affect and larger decreases in low‐arousal negative affect after spending 10 min alone (compared to the Control condition).

To explore these questions, we used methods nearly identical to those of Rodriguez et al. (2020), with a few key changes described in detail below.

## 3 METHOD

All procedures received institutional approval from the Harvard University Committee on the Use of Human Subjects (IRB20‐0590). The Stage 1 Registered Report, recruitment materials, stimuli, R code, and de‐identified dataset are publicly available online at an Open Science Framework page created for this study: https://osf.io/rgcde/.

### 3.1 Participants

Harvard undergraduates and community members from the Boston‐Cambridge area were recruited via the Harvard University Department of Psychology's research website. The website advertised the study under the title “Social Media, Emotions, and You” and did not mention either loneliness or solitude in the study description. Participants were recruited during the Fall 2021, Spring 2022, Summer 2022, and Fall 2022 semesters. In Fall 2021 and Spring 2022, participants received either USD $10 in cash or 1.0 psychology study pool credits for their time and effort. Due to slow enrollment attributable to the COVID‐19 pandemic and the proliferation of online studies, compensation was increased to USD $20 in cash or 2.0 psychology study pool credits for the Summer and Fall of 2022 to incentivize participation.

Inclusion criteria comprised fluency in English, being at least 18 years of age, and reporting moderate‐to‐severe levels of loneliness. To assess loneliness, we asked prospective participants to complete a pre‐screening survey directly on the research recruitment website. The pre‐screening survey consisted of the UCLA Loneliness Scale, 8‐item version (ULS‐8; Hays & DiMatteo, 1987). As our study targets people high in trait loneliness, prospective participants were required to score at least one standard deviation above the mean of loneliness on the ULS‐8 pre‐screener, as assessed in a prior study that utilized a general adult sample from the same geographic area (i.e., a score of 21 or greater on a 4 to 32 scale; Rodriguez et al., 2020), in order to participate.

In total, 807 community members and 807 undergraduate students completed the pre‐screener. Of these, 274 community members (34.0%) and 164 undergraduate students (20.3%) met eligibility criteria and were subsequently contacted via email to sign up for an in‐person appointment at our laboratory. A total of 112 community members and 112 undergraduate students completed the in‐person laboratory session. All community members received monetary compensation for their participation, whereas undergraduate students received psychology study pool credits.

### 3.2 Measures

**FIGURE 1:**
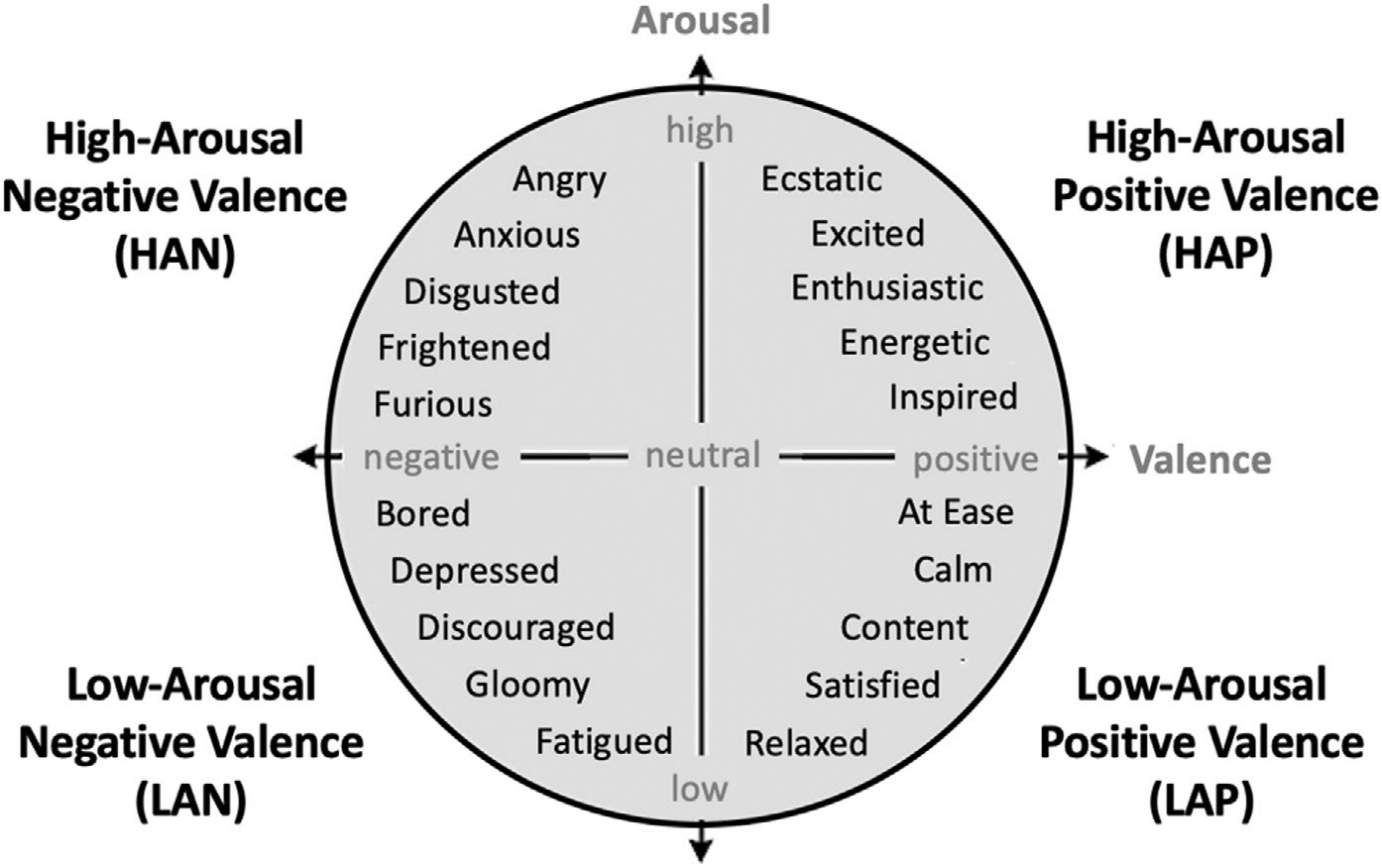
Affective states assessed in this study by valence and arousal. *Source*: Adapted from Posner et al. (2005).

*State affect* was measured via the Job‐Related Affective Wellbeing Scale (JAWS; van Katwyk et al., 2000). Here, we deviate from our previous method (Rodriguez et al., 2020), where we used the Positive and Negative Affect Schedule (PANAS; Watson et al., 1988) to assess affect. A major limitation of the PANAS is that it primarily measures high‐arousal affective states (e.g., *enthusiastic*; *distressed*), thus preventing our ability to capture changes in other pertinent dimensions of affect. As solitude tends to be a low‐arousal activity, our ability to measure low‐arousal mood states (e.g., *calm; gloomy*) is imperative. We selected the JAWS because it captures the four quadrants of emotion outlined in the circumplex model of affect (Russell, 1980): high‐arousal positive (HAP; e.g., *ecstatic*), low‐arousal positive (LAP; e.g., *relaxed*), high‐arousal negative (HAN; e.g., *angry*), and low‐arousal negative (LAN; e.g., *bored*; See Figure 1). Participants rated the degree to which they felt 20 emotional states in the present moment (e.g., “I feel anxious”) on a 5‐point Likert scale (1 = *not at all*, 5 = *extremely*). The JAWS was administered twice—before and after the experimental manipulation and solitude period—to capture changes in affect. All four subscales displayed adequate to very good internal consistency at both time points [HAP: *α* = 0.84 (Time 1), *α* = 0.86 (Time 2); LAP: *α* = 0.87 (Time 1), *α* = 0.91 (Time 2); HAN: *α* = 0.69 (Time 1), *α* = 0.71 (Time 2); LAN: *α* = 0.78 (Time 1), *α* = 0.70 (Time 2)].

*Trait loneliness* was measured using the UCLA Loneliness Scale–8‐item version (ULS‐8; Hays & DiMatteo, 1987). Participants rated the extent to which they felt isolated from others in everyday life (e.g., “There is no one I can turn to”) on a 4‐point Likert scale (1 = *never*, 4 = *often*). The ULS‐8 was administered twice: once during the pre‐screening survey and again during the in‐person laboratory session. This scale demonstrated adequate internal consistency in our sample (*α* = 0.79).

*Trait reappraisal*, which refers to individuals' tendency to use cognitive reappraisal in daily life, was assessed using the Emotion Regulation Questionnaire–Reappraisal subscale (ERQ; Gross & John, 2003). Participants rated their level of agreement with six statements (e.g., “When I want to feel less negative emotion, I change the way I'm thinking about the situation”) on a 7‐point Likert scale (1 = *strongly disagree*, 7 = *strongly agree*). Due to a data collection error, the Qualtrics survey recorded trait reappraisal data for only 82 participants (i.e., 38% of our final sample used in analyses). Thus, any analyses that include trait reappraisal may be substantially underpowered to detect effects if they exist. This scale had adequate internal consistency in our sample (*α* = 0.73).

*Perceptions of inferiority on social media* were assessed via the Social Comparison Scale (Allan & Gilbert, 1995). This scale consists of 11 pairs of opposing constructs (e.g., *inferior* vs. *superior*; *insider* vs. *outsider*; *unattractive* vs. *very attractive*). Participants rated how they felt about themselves in relation to others on a continuum between pairs of constructs on a scale of 1 (*highest rating on the first extreme of the construct*) to 10 (*highest rating on the second extreme of the construct*). Total scores were reverse coded such that higher scores indicate greater self‐perceptions of inferiority compared with others, whereas lower scores indicate lower self‐perceptions of inferiority compared with others. This scale demonstrated very good internal consistency in our sample (*α* = 0.88).

*Compulsive social media use* was assessed via a modified version of the Compulsive Internet Use Scale (Meerkerk et al., 2009). We adapted this scale to ask specifically about social media use, rather than internet use writ large (Rodriguez et al., 2020). This scale assesses the severity of compulsive, pathological, and problematic behaviors when using social media, as well as the degree to which such behaviors interfere with everyday functioning. Participants rated their level of agreement with eight items (e.g., *How often have you unsuccessfully tried to spend less time on social media?*) on a 6‐point Likert scale (1 = *never*, 6 = *very often*). This scale had good internal consistency in our sample (α = 0.81).

#### 3.2.1 Solitude period questionnaire

First, participants reported the degree to which the passage they read challenged their beliefs about solitude on a 5‐point Likert scale (1 = *my beliefs were not challenged at all*, 5 = *my beliefs were extremely challenged*)—this served as a manipulation check. Next, participants indicated the extent to which they felt lonely during the 10 min solitude period (1 = *not lonely at all*, 5 = *extremely lonely*). Participants also indicated the extent to which they felt anxious without their phones (1 = *strongly disagree*, 5 = *strongly agree*) and the extent to which they felt uncomfortable with their thoughts (1 = *strongly disagree*, 5 = *strongly agree*) during the solitude period. Finally, participants reported the extent to which they experienced the 10 min solitude period positively (1 = *not positively at all*, 5 = *extremely positively*) and negatively (1 = *not negatively at all*, 5 = *extremely negatively*). Please note that we refer to this questionnaire as “Solitude Activities” in our Stage 1 Registered Report.

#### 3.2.2 Demographic information

Participants indicated their age, gender, race/ethnicity, and highest level of educational attainment.

### 3.3 Experimental manipulation

Participants were randomly assigned to read one of three brief informational passages. Passages were standardized in length (195–205 words) and content (e.g., all contained statistical data to support their claims). These passages were identical to those used in our team's original experiment (Rodriguez et al., 2020). Please see a description of each passage below:

1. *Solitude Benefits* passage frames time alone as a positive experience by describing its various benefits (e.g., mental restoration, stress reduction, and creativity). The *Solitude Benefits* passage aims to help individuals frame solitude as beneficial to their well‐being rather than view it as an inherently lonely experience.
2. *Loneliness De‐Biasing* passage normalizes loneliness by framing it as a normal part of the human experience. This passage describes the widespread prevalence of loneliness in the American population. The *Loneliness De‐Biasing* passage also aims to help people reframe solitude, but via a different mechanism: by challenging the belief that loneliness is rare and indicative of personal inadequacy.
3. *Control* passage describes the Harvard University Psychology Study Pool, an online platform that allows individuals to participate in psychological research. The *Control* passage does not mention “loneliness,” “solitude,” or “time alone.”

### 3.4 Procedure

#### 3.4.1 Pre‐screening questionnaire

As previously mentioned, individuals interested in our study were first required to complete a brief online pre‐screening questionnaire assessing trait levels of loneliness (i.e., the ULS‐8). Eligible participants (i.e., those who scored 21 or above on the ULS‐8) were subsequently contacted via email or phone to sign up for an in‐person appointment at our laboratory.

#### 3.4.2 Informed consent

Upon arrival at the laboratory, participants were greeted by the experimenter and were asked to leave all personal belongings and electronic devices (e.g., cellphones, smartwatches, laptops, wearable fitness trackers) in a separate room. Once in the testing room, participants read and signed an institutionally approved consent form with detailed information about the 45‐min laboratory study and the specific activities involved.

#### 3.4.3 Baseline measures

After providing informed consent, participants completed a battery of baseline measures on an online Qualtrics survey administered from a laboratory computer (i.e., trait loneliness, compulsive social media use, perceptions of inferiority, and trait reappraisal). Participants also reported their state affect.

#### 3.4.4 Experimental manipulation

Next, participants were randomly assigned to read one of three informational passages (*Solitude Benefits*, *Loneliness De‐Biasing*, or *Control*).

#### 3.4.5 Solitude period

Participants were then informed that there would be a “waiting period” of approximately 10 min “while the survey processes their responses.” The true purpose of the waiting period—to examine whether reframing beliefs about being alone shapes the emotional effects of solitude—was masked to participants. The Qualtrics survey automatically began a 10‐min timer and advanced to the next page after 10 min had elapsed.

#### 3.4.6 Post‐solitude period questionnaires

After the “waiting period,” participants reported their state affect once again. Participants then completed the Solitude Period Questionnaire and provided demographic information.

#### 3.4.7 Debriefing

Lastly, the experimenter fully debriefed participants about the purpose of the experiment and gave participants a debriefing form to take home. Participants were thanked for their time and were compensated.

### 3.5 Planned analyses

We conducted all analyses with R version 1.4.1717 (R Core Team, 2021).

#### 3.5.1 Power analysis

An a priori power analysis indicated that a sample of *N* = 243 participants is required to achieve sufficient power (1 − *β* error probability = 0.80) to detect a small effect size in our analyses of variance (ANOVA). Our target effect size ($\eta_{p}^{2}$ = 0.03; small effect) was analogous to the effect size found in our prior experiment (Rodriguez et al., 2020).

#### 3.5.2 Exclusion criteria

Before analyzing data, we planned to exclude any participants who: (a) called in the experimenter or left the laboratory room during the solitude period; (b) smuggled a personal electronic device (e.g., cellphone, smartwatch) into the laboratory room; (c) used the laboratory computer to surf the web during the solitude period; (d) fell asleep during the solitude period; or (e) had multiple instances of missing data. After approval of our Stage 1 Registered Report, we realized that criterion (e) was rather vague. Thus, prior to conducting analyses, we decided to exclude participants who had missing data for 10% or more of survey questions.

#### 3.5.3 Missing data

We planned to use mean imputation for single instances of missing responses (e.g., one item from a measure), as we did in our prior experiment (Rodriguez et al., 2020). For participants with more than one missing item within the same measure, we planned to omit their data for that measure. We planned to exclude from analyses any participant who did not respond to at least 10% of the survey questions.

#### 3.5.4 Outlier detection procedure

Before conducting analyses, we planned to perform several procedures to detect extreme outliers, including visual inspection of boxplots, Cook's distance calculations, and standard deviation calculations (i.e., ± 3 *SD*s from the mean). We planned to report any outliers and run all our models with and without the outliers.

#### 3.5.5 Preliminary analyses

First, we planned to examine sample characteristics (e.g., age, gender, race/ethnicity, education level) and conduct condition‐wise group comparisons on these variables to test whether random assignment resulted in conditions that did not differ from one another in key demographic characteristics. Next, we planned to conduct a series of bivariate correlations (Pearson's *r*) to assess the zero‐order associations between trait loneliness, trait reappraisal, perceptions of inferiority on social media, compulsive social media use, and participant age. We also planned to examine whether each of the four quadrants of affect (i.e., HAN, HAP, LAN, and LAP) significantly changed during the solitude period via a series of independent samples *t*‐tests. Finally, as a manipulation check, we planned to conduct an ANOVA to examine whether conditions (*Solitude Benefits*, *Loneliness De‐Biasing*, and *Control*) differed in the extent to which they challenged participants' beliefs about solitude.

#### 3.5.6 Main effects

To test Hypothesis 1, we planned to calculate change scores for each of the four quadrants of affect: low‐arousal positive (LAP), high‐arousal positive (HAP), low‐arousal negative (LAN), and high‐arousal negative (HAN). Change scores (*T*_2_ affect − *T*_1_ affect) reflect the change in affect resulting from the experimental manipulation and solitude period. Then, we planned to conduct two ANOVAs to determine whether changes in LAP or changes in LAN significantly differed between the experimental conditions and *Control*. We planned to adjust our *p* values for the false discovery rate (FDR; Benjamini & Hochberg, 1995). In the case of significant differences, we planned to conduct Tukey's post hoc tests to determine the direction and magnitude of these differences. We planned to report both Bonferroni‐corrected and uncorrected results in our manuscript.

#### 3.5.7 Moderation effects

To test Hypothesis 2, we planned to conduct a series of regression‐based interaction tests to determine whether trait reappraisal, perceptions of inferiority, or compulsive social media use moderated the effect of the experimental manipulations on changes in LAP and LAN. For each multiple regression, we planned to include condition as a dummy‐coded independent variable (*Control* = 0, *Loneliness De‐Biasing* = 1, *Solitude Benefits* = 2), the proposed moderator as a continuous independent variable, and the cross‐product of condition and the proposed moderator as an independent variable. We planned to adjust our *p* values in this analysis for the FDR correction and report both corrected and uncorrected analyses. In the case of any statistically significant cross‐product terms, we planned to conduct a simple slopes analysis to interpret the effects of condition on changes in affect at varying levels of the moderator.

## 4 RESULTS

### 4.1 Sample characteristics

Two hundred and twenty‐four participants completed our study. Exactly half of the participants were Harvard undergraduate students (*n* = 112), and the other half were community members from the Boston‐Cambridge area (*n* = 112). Twelve participants were excluded for either calling the experimenter in during the solitude period (*n* = 3), falling asleep during the solitude period (*n* = 5), surfing the web on the laboratory computer during the solitude period (*n* = 3), or smuggling their cellphone into the laboratory room (*n* = 1).

The final sample comprised 212 participants. Their mean age was 25.59 years old (*SD* = 9.87, range: 18–69). Most participants identified as female (*n* = 138, 65.1%), and the rest identified as either male (*n* = 66, 31.1%) or non‐binary (*n* = 8, 3.8%). Participants identified their race or ethnicity as either White (*n* = 85, 40.1%), Asian (*n* = 73, 34.4%), Black (*n* = 32, 15.1%), Latino (*n* = 18, 8.5%), Native Hawaiian/Pacific Islander (*n* = 1, 0.5%), or Other (*n* = 3, 1.4%). All participants had at least a high school diploma, and many had completed some college (or were currently enrolled in college; *n* = 108, 50.9%), attained a bachelor's degree (*n* = 44, 20.8%), attained a master's degree (*n* = 30, 14.2%), or attained a professional or doctoral degree (*n* = 6, 2.8%).

All participants indicated moderate‐to‐severe levels of loneliness on the pre‐screening questionnaire. When loneliness was assessed again during the main laboratory study, the mean score was 22.46 (*SD* = 4.26), which remains above the criterion for inclusion. Of note, a subsample of participants (*n* = 61, 28.77%) no longer met the initial inclusion criteria (i.e., scored below 21 on the ULS‐8) at the time of the laboratory session, suggesting that their loneliness levels decreased between taking the online pre‐screening questionnaire and completing the study. At the suggestion of an anonymous reviewer, we decided post hoc to re‐run the main analyses and any statistically significant moderator analyses on the subsample of individuals (*n* = 151) who still met the pre‐specified threshold for loneliness at the time of the laboratory session.

The Qualtrics survey randomly assigned participants to either the *Solitude Benefits* condition (*n* = 66), the *Loneliness De‐Biasing* condition (*n* = 72), or the *Control* condition (*n* = 74). Condition‐wise group comparisons revealed that neither age, gender, race, education level, nor trait loneliness significantly differed across the three conditions (*Solitude Benefits*, *Loneliness De‐Biasing*, or *Control*).

### 4.2 Bivariate correlations

Table 1 displays bivariate correlations between age, trait loneliness, compulsive social media use, perceptions of inferiority, and trait reappraisal. Notably, lonelier people more compulsively used social media, perceived themselves as more inferior to others, and used cognitive reappraisal less to regulate their emotions in daily life. Younger people in our sample were more likely to compulsively use social media and to perceive themselves as inferior to others than older people.

**TABLE 1:** Descriptive statistics and bivariate correlations between trait variables (*N* = 212).

	*M* (SD)	Age	Loneliness	CSMU	Inferiority
Loneliness	22.46 (4.26)	0.05			
Compulsive SMU	24.15 (6.13)	−0.27	0.15*		
Inferiority	70.77 (15.14)	−0.16*	0.32***	0.12	
Trait reappraisal	21.04 (4.25)	−0.01	−0.26**	−0.11	–0.15

* *p* < 0.05

** *p* < 0.01

*** *p* < 0.001.

### 4.3 Changes in affect across conditions

First, we examined whether four types of affect (i.e., LAP, HAP, LAN, and HAN) significantly changed after 10 min of solitude. Across all three conditions, LAP significantly increased, *t*(211) = 3.18, *M*_Diff_ = 0.74, *p* = 0.0017, *d* = 0.22 (small effect); HAP significantly decreased, *t*(211) = −7.05, *M*_Diff_ = −1.36, *p* < 0.001, *d* = −0.48 (medium‐to‐large effect); LAN did not significantly change, *t*(211) = 0.32, *M*_Diff_ = 0.06, *p* = 0.75; and HAN significantly decreased, *t*(211) = −7.69, *M*_Diff_ = −0.85, *p* < 0.001, *d* = −0.53 (medium‐to‐large effect).

We then examined changes in affect separately for each condition (see Table 2). LAP significantly increased in the *Solitude Benefits* and *Loneliness De‐Biasing* conditions, but not in the *Control* condition. HAP significantly decreased in all three conditions. LAN did not significantly change in any of the three conditions. HAN significantly decreased in all three conditions.

**TABLE 2:** Mean changes in affect after the 10‐min solitude period by condition.

Condition	Change in positive affect		Change in negative affect	
	Low‐arousal (LAP)	High‐arousal (HAP)	Low‐arousal (LAN)	High‐arousal (HAN)
*Solitude Benefits*	1.12* Increase	−1.12** Decrease	−0.18 No change	−0.85*** Decrease
*Loneliness De‐Biasing*	1.00** Increase	−1.17*** Decrease	0.14 No change	−1.01*** Decrease
*Control*	0.15 No change	−1.76*** Decrease	0.19 No change	−0.69** Decrease

* *p* < 0.05

** *p* < 0.01

*** *p* < 0.001.

### 4.4 Manipulation check

Both the *Solitude Benefits* (*M* = 2.87, *M*_Diff_ = 0.54, *p* = 0.007) and *Loneliness De‐Biasing* (*M* = 2.74, *M*_Diff_ = 0.41, *p* = 0.046) conditions challenged participants' beliefs about solitude more strongly than the *Control* condition (*M* = 2.32); *F*(2, 209) = 5.24, *p* = 0.006.

### 4.5 Main effects

#### 4.5.1 Changes in low‐arousal positive affect (Hypothesis 1A)

**FIGURE 2:**
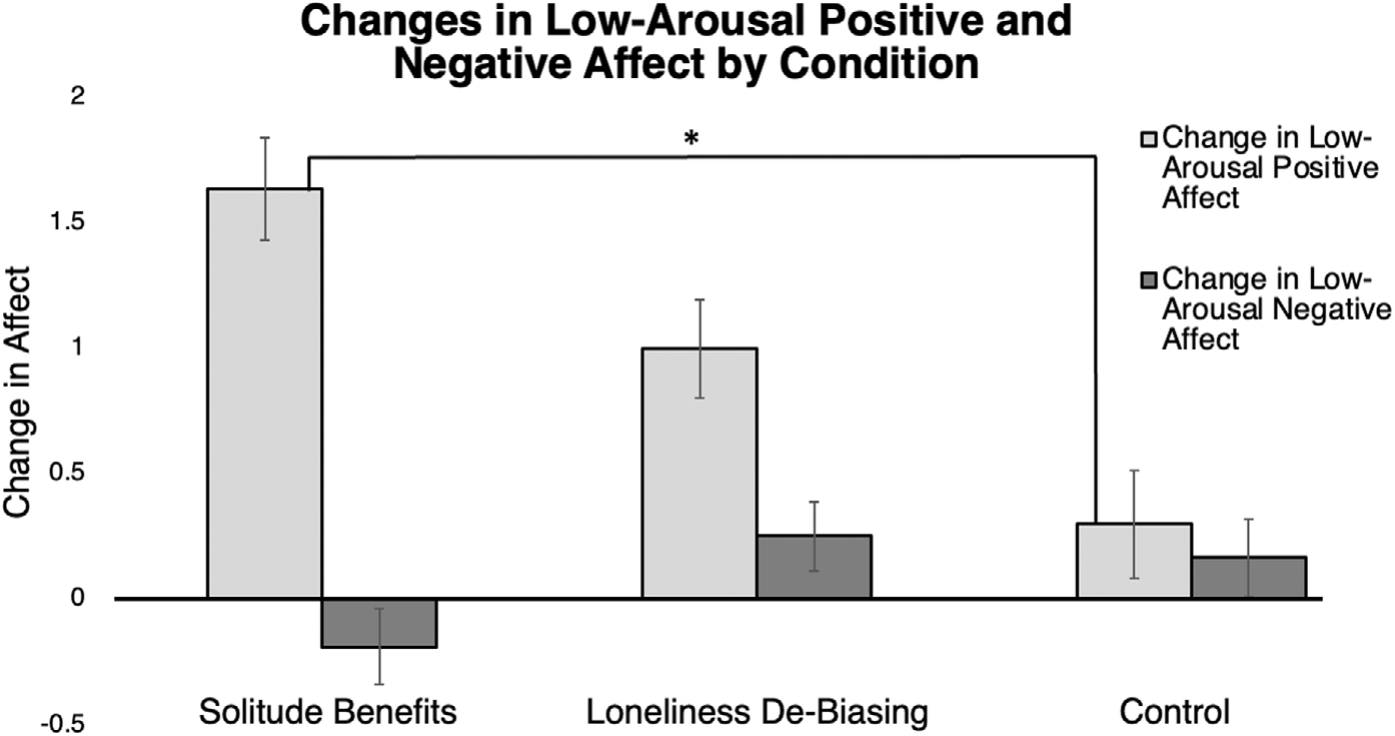
Changes in low‐arousal positive and negative affect by condition. Asterisk (*) indicates *p* < 0.05.

Our outlier detection procedures detected four outliers in our dataset; we excluded these participants. There was a small effect of condition on changes in LAP; *F*(2, 205) = 3.28, *p* = 0.04; $\eta_{p}^{2}$  = 0.0.03. A post hoc analysis with the Tukey adjustment revealed that *Solitude Benefits* participants experienced a larger increase in LAP (*M* = 1.63) after the solitude period than *Control* participants (*M* = 0.30; *M*_Diff_ = 1.33, *p* = 0.03). Although *Loneliness De‐Biasing* participants also experienced an increase in LAP after the solitude period (*M* = 1.00), this increase was not significantly different from *Solitude Benefits* (*M*_Diff_ = −0.63, *p* = 0.45) or *Control* (*M*_Diff_ = 0.70, *p* = 0.35). Figure 2 displays a graph of changes in LAP by condition. We reran the model with the four outliers and report these results in the footnote below.^1^ This result holds when analyzing the subsample of participants (*n* = 151) who met the pre‐specified threshold for loneliness at the time of the laboratory session.^2^

#### 4.5.2 Changes in low‐arousal negative affect (Hypothesis 1B)

Our outlier detection procedure detected five outliers, which were removed. There was no significant effect of condition on changes in LAN; *F*(2, 204) = 0.74, *p* = 0.48. We reran the model with the five outliers and report the results below.^3^ This result holds in the subsample of participants who met the pre‐specified threshold for loneliness at the time of the laboratory session.^4^ Figure 2 displays a graph of changes in LAN by condition.

### 4.6 Moderation analyses

#### 4.6.1 Trait reappraisal (Hypothesis 2A)

The regression to detect an interaction between trait reappraisal and condition on change in LAP did not yield significant cross‐products between trait reappraisal and either the *Solitude Benefits* condition, *t*(74) = 0.37, *p* = 0.72, or the *Loneliness De‐Biasing* condition, *t*(74) = 0.19, *p* = 0.85 *F*(5, 74) = 0.85, *p* = 0.52, *R* ^2^ = 0.05. The regression to detect an interaction between trait reappraisal and condition on change in LAN also did not yield significant cross‐products between trait reappraisal and either the *Solitude Benefits* condition, *t*(75) = 0.01, *p* = 0.99, or the *Loneliness De‐Biasing* condition, *t*(75) = − 0.78, *p* = 0.44; *F*(5, 75) = 1.50, *p* = 0.20, *R* ^2^ = 0.03.

Notably, we were likely underpowered to detect an interaction effect due to the error with collecting trait reappraisal data described under *Methods*. We report the results of the model including the four outliers here.^5^

#### 4.6.2 Perceptions of inferiority (Hypothesis 2B)

The regression to detect an interaction between perceptions of inferiority on social media and condition on change in LAP yielded significant cross‐products between perceptions of inferiority and the *Solitude Benefits* condition, *t*(202) = −2.48, *p* = 0.014, but not the *Loneliness De‐Biasing* condition, *t*(202) = −0.79, *p* = 0.43; *F*(5, 202) = 3.59, *p* = 0.004, *R* ^2^ = 0.08. This interaction effect remained significant when we controlled for the FDR correction (adjusted *p* is 0.05/3 or 0.017).

**FIGURE 3:**
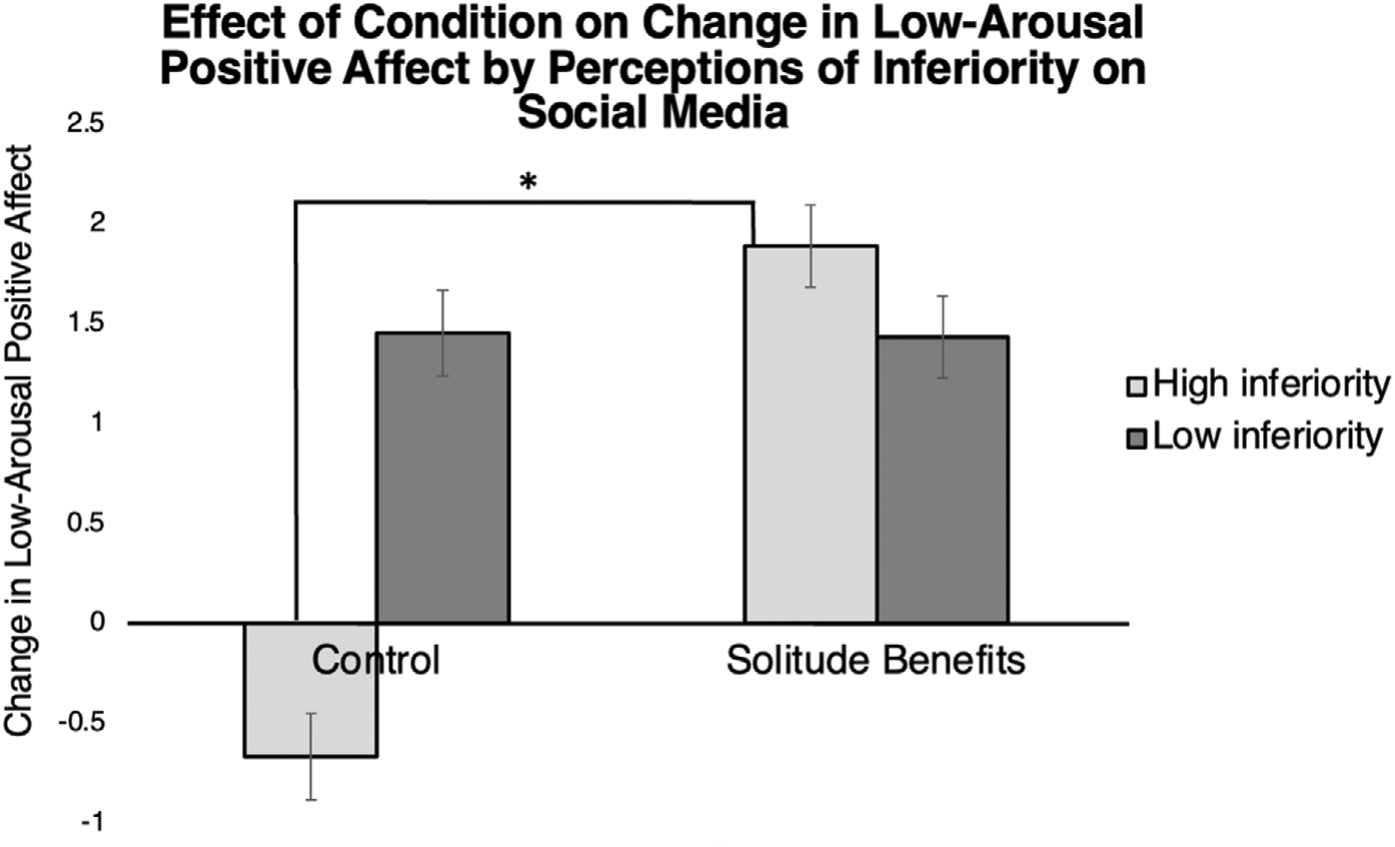
Effect of condition on change in low‐arousal positive affect by perceptions of inferiority on social media. Asterisk (*) indicates *p* < 0.05.

To examine this interaction further, we conducted a simple slopes analysis to determine the effect of condition on change in LAP at one *SD* above and one *SD* below the mean level of perceptions of inferiority on social media (see Figure 3). For individuals who perceive themselves as highly inferior to others on social media, the *Solitude Benefits* condition produced a significantly larger increase in LAP compared with *Control*, *t*(204) = 3.44, *p* < 0.001, *b* = 1.28. For those who did not perceive themselves as inferior to others on social media, the *Solitude Benefits* condition did not significantly change LAP compared with *Control*, *t*(204) = −0.03, *p* = 0.977, *b* = −0.10. We report the results of the model with the four outliers here.^6^ These results hold in the subsample of participants who met the pre‐specified threshold for loneliness at the time of the laboratory session.^7^

The regression to detect an interaction between perceptions of inferiority on social media and condition on change in LAN did not yield significant cross‐products between perceptions of inferiority on social media and either the *Solitude Benefits* condition, *t*(201) = 0.11, *p* = 0.92 or the *Loneliness De‐Biasing* condition, *t*(201) = 0.64, *p* = 0.53; *F*(5, 201) = 1.54, *p* = 0.18, *R* ^2^ = 0.01.

#### 4.6.3 Compulsive social media use (Hypothesis 2C)

The regression to detect an interaction between compulsive social media use and condition on change in LAP did not yield significant cross‐products between compulsive social media use and either the *Solitude Benefits* condition, *t*(202) = −0.54, *p* = 0.59 or the *Loneliness De‐Biasing* condition, *t*(202) = 1.67, *p* = 0.097; *F*(5, 202) = 2.28, *p* = 0.048, *R* ^2^ = 0.05. We report results of the model including the four outliers here.^8^

The regression to detect an interaction between compulsive social media use and condition on change in LAN did yield significant cross‐products between compulsive social media use and the *Loneliness De‐Biasing* condition, *t*(201) = −2.83, *p* = 0.005, but not the *Solitude Benefits* condition, *t*(201) = −0.27, *p* = 0.79, *F*(5, 201) = 2.31, *p* = 0.05, *R* ^2^ = 0.03. This interaction effect remained significant when controlling for FDR correction (adjusted *p* is 0.05/3 or 0.017). We report the results of the model with the four outliers here.^9^ This result does not hold in the subsample of participants who met the pre‐specified threshold for loneliness at the time of the laboratory session.^10^

**FIGURE 4:**
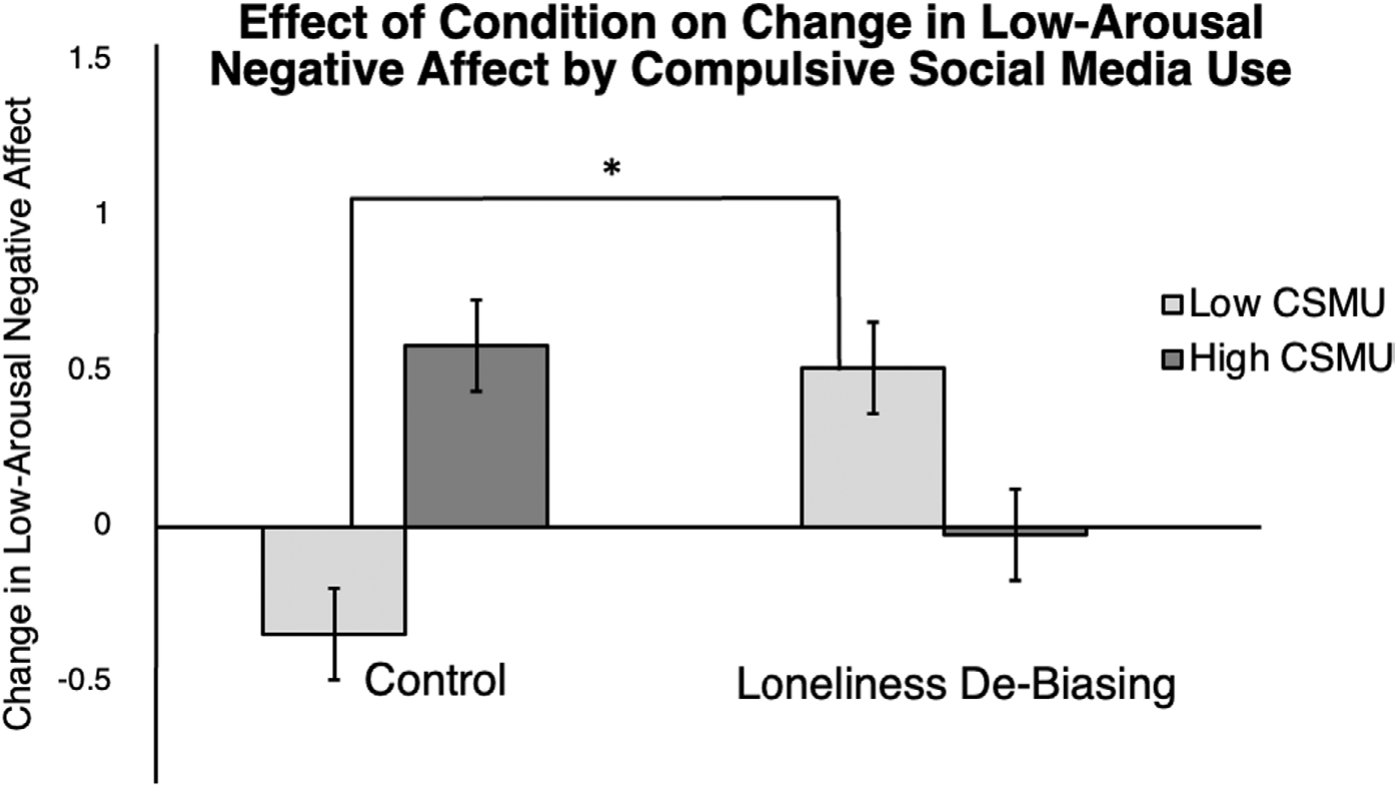
Effect of condition on change in low‐arousal negative affect by compulsive social media use. Asterisk (*) indicates *p* < 0.05.

To examine this interaction further, we conducted a simple slopes analysis to determine the effect of condition on change in LAN at one *SD* above and one *SD* below the mean level of compulsive social media use (see Figure 4). For individuals who do not compulsively use social media, the *Loneliness De‐Biasing* condition produced an increase in LAN, whereas the *Control* condition produced a decrease in LAN, *t*(204) = 2.23, *p* = 0.028, *b* = 1.12. For people who more compulsively use social media, the *Loneliness De‐Biasing* condition did not significantly change LAN compared with the *Control* condition, *t*(204) = −1.88, *p* = 0.063, *b* = −0.95.

### 4.7 Exploratory analyses

In addition to the planned analyses described in our Stage 1 Registered Report, we conducted exploratory analyses to investigate the following questions: First, does condition significantly affect feelings of state loneliness during the solitude period? Additionally, who are the people who felt loneliest during the 10‐min solitude period? To address this, we examined whether, and the extent to which, both condition and individual differences predicted how lonely people felt during the solitude period (Exploratory Analysis 1). Second, given possible floor effects in LAN change, we further probed Hypothesis 1B by examining whether condition affects how negatively participants self‐report experiencing the solitude period (Exploratory Analysis 2).

#### 4.7.1 Exploratory analysis 1: Does condition and/or individual differences predict how lonely people felt during the solitude period?

We conducted a series of exploratory analyses to examine whether condition and/or individual differences predicted feelings of loneliness during the solitude period. We controlled for trait loneliness in these exploratory analyses to examine how variables of interest predict state loneliness above and beyond trait levels of loneliness.

First, we ran an ANOVA to examine whether condition affected how lonely people felt during the solitude period. There was no significant effect of condition on loneliness during the solitude period; *F*(2, 208) = 2.75, *p* = 0.09. Then, we ran a multiple regression to examine whether individual differences predicted how lonely people felt during the solitude period (see Table 3). We found that people who were higher in trait loneliness felt lonelier during the solitude period; people who felt more anxious without their phone felt lonelier during the solitude period; and people who were uncomfortable with their thoughts felt lonelier during the solitude period. Compulsive social media use and perceptions of inferiority did not significantly predict feelings of loneliness during the solitude period.

**TABLE 3:** Multiple regression testing predictors of state loneliness during the solitude period.

	*b*	*SE*	*t*	*p*
Trait loneliness	0.04	0.02	2.33	0.021
Compulsive social media use	0.01	0.01	0.74	0.461
Perceptions of inferiority	0.002	0.005	0.31	0.757
Anxious without phone	0.15	0.07	2.20	0.029
Uncomfortable with thoughts	0.26	0.07	3.97	0.000

Abbreviations: *b*, unstandardized beta coefficient; *SE*, standard error.

#### 4.7.2 Exploratory analysis 2: Does condition affect the extent to which participants experienced the solitude period negatively?

We found that *Solitude Benefits* participants (*M* = 1.53) experienced their solitude significantly less negatively than did Control participants, *M* = 2.03, *M*_Diff_ = −0.50, *F*(2, 209) = 5.13, *p* = 0.007; $\eta_{p}^{2}$ = 0.05. There was no significant difference between the *Loneliness De‐Biasing* condition (*M* = 1.74) and either the *Control* (*M*_Diff_ = −0.29, *p* = 0.14) or *Solitude Benefits* (*M*_Diff_ = 0.21, *p* = 0.39) conditions.

## 5 DISCUSSION

Prior research from our team has found that reframing solitude as beneficial to one's health helps individuals preserve positive affect when alone (Rodriguez et al., 2020). Here, we build on this work by (a) testing whether our findings replicate in a sample of individuals experiencing moderate‐to‐severe loneliness and (b) assessing low‐arousal and high‐arousal affect separately. These modifications allowed us to determine whether Rodriguez et al.'s (2020) findings extend to a more vulnerable group (i.e., lonelier people) who struggle in solitude and may benefit more from reframing their time alone. These changes also enabled us to capture the effects of reframing solitude across both low‐arousal and high‐arousal states. Notably, this study demonstrates that people who are lonely experience a larger increase in low‐arousal positive emotions (e.g., calm, contentment) during a brief period of solitude when they reframe solitude as beneficial to their well‐being. Below, we discuss each set of findings and their potential implications.

### 5.1 What are the emotional effects of a brief period in solitude among people who are lonely?

Participants across conditions experienced substantial reductions in high‐arousal positive affect (e.g., *excitement*) and high‐arousal negative affect (e.g., *anger*) after only 10 min of solitude. This finding is consistent with research suggesting that solitude serves as an arousal “deactivator” by reducing the intensity of high‐arousal positive and negative emotions (Nguyen et al., 2018). Our findings are notable for two reasons. First, although the benefits of chosen (or self‐determined) solitude are well‐established (e.g., Lay et al., 2020; Tse et al., 2022), our research suggests that even unchosen solitude can deactivate intense emotions and promote affective regulation. Second, we show that even people struggling with high levels of loneliness—who generally view solitude as an aversive experience—can experience solitude more positively by altering the way they think about being alone.

### 5.2 Does cognitively reframing solitude impact its emotional effects among people who are lonely?

#### 5.2.1 Hypothesis 1A

Participants who read about the benefits of solitude (i.e., *Solitude Benefits* condition) experienced a greater increase in low‐arousal positive affect after spending 10 min alone compared to those who read a control passage unrelated to solitude. In other words, positively reframing solitude enabled lonely individuals to experience more positive feelings, like contentment and relaxation, when physically alone. Importantly, our data suggest that cognitive change may be the mechanism responsible for this effect, as reading about the benefits of solitude challenged participants' beliefs significantly more than the control passage. Thus, our findings suggest that people experiencing loneliness experience solitude more positively when they reframe solitude as a beneficial experience that promotes their health and well‐being.

Reading about the high prevalence of loneliness (i.e., *Loneliness De‐Biasing* condition) did not significantly impact changes in low‐arousal positive affect compared with the *Control* or *Solitude Benefits* conditions. Although the *Loneliness De‐Biasing* passage challenged participants' beliefs about solitude more than *Control* passage, it did not provide the same emotional boost as reading about the benefits of solitude. One possible explanation is that the *Loneliness De‐Biasing* passage made loneliness more salient to participants who were already struggling with loneliness. Although the passage aimed to de‐stigmatize loneliness, it may have inadvertently primed participants to think about their loneliness during the subsequent solitude period. Another possibility is that participants were already aware of the high prevalence of loneliness because it is so widely discussed in the media, particularly in the aftermath of the COVID‐19 pandemic (Murthy, 2021; Smith et al., 2023). Regardless, our finding aligns with Rodriguez et al.'s (2020) conclusion that reading about the benefits of solitude, but not de‐stigmatizing loneliness, helps people experience their time alone more positively. Our findings thus provide partial support for Hypothesis 1A.

It is noteworthy that such a simple manipulation (i.e., reading a 200‐word passage about the benefits of solitude) was sufficient to improve participants' emotional experience during solitude. As mentioned earlier, this may be because  people experiencing loneliness initially endorse more negative beliefs about solitude (Rodriguez et al., 2020). Specifically, individuals who are lonely may perceive solitude as aversive, painful, and a reminder of their social isolation (Smith & Victor, 2019; Weinstein et al., 2021). In this case, even a slight suggestion that solitude can be an enjoyable experience may be impactful for these individuals. More broadly, this study adds to a large body of work on the power of simple and brief cognitive interventions to effectively improve our responses to potentially stressful situations (e.g., growth mindset and stress mindset interventions; Crum et al., 2017; Yeager et al., 2019).

This study provides insight into the replicability of Rodriguez et al.'s (2020) study on reframing time alone. Rodriguez et al. (2020) found that spending 10 min in solitude “deactivated” both high‐arousal positive and negative affect across conditions, which is consistent with our current findings. Furthermore, in both Rodriguez et al. (2020) and the present study, the *Loneliness De‐Biasing* condition did not significantly differ from either the *Control* condition or the *Solitude Benefits* condition. Finally, the statistical size of the effects (i.e., small) is identical between the two studies.

Both the present study and Rodriguez et al.'s (2020) original study found that reading about the benefits of solitude, compared to control, significantly improves emotional experiences during solitude. However, while Rodriguez et al. (2020) found that reading about the benefits of solitude merely *buffered* against a decrease in positive affect, the present study found that it *increased* low‐arousal positive affect among people experiencing loneliness. One possible reason that the effects differed across studies is that affect was measured using different instruments. Rodriguez et al. (2020) assessed affect using the Positive and Negative Affect Schedule (PANAS; Watson et al., 1988). The PANAS is a widely used instrument; however, it is restricted to mostly high‐arousal positive and negative states (Roca et al., 2023; Tellegen et al., 1999). As a result, the PANAS is not ideal for assessing the emotional effects of solitude, given that solitude is a low‐arousal activity that differentially affects low‐ and high‐arousal affective states (Pauly et al., 2017). In the current study, we measured both low‐arousal and high‐arousal affective states, which enabled a more granular assessment of the emotional effects of solitude. Another possible reason for the differences between studies is that our present sample of people experiencing moderate‐to‐severe loneliness differed meaningfully from the general adult sample in our original experiment. It is unknown whether the difference between our results and those of Rodriguez et al. (2020) is due to differences in how affect was measured, differences in the samples studied, or a combination of the two.

#### 5.2.2 Hypothesis 1B

Contrary to our hypotheses, but in line with Rodriguez et al. (2020), neither the *Solitude Benefits* nor the *Loneliness De‐Biasing* manipulations significantly affected changes in low‐arousal negative affect relative to *Control*. People who read about the benefits of solitude experienced a slight decrease in low‐arousal negative affect (e.g., *depressed*; *bored*) after 10 min of solitude, but this change was not significantly different from the other conditions. This result may be due to floor effects in low‐arousal negative affect. On average, participants reported lower levels of low‐arousal negative affect at baseline than they did low‐arousal positive affect. Thus, it is possible that the low base rate of low‐arousal negative affect rendered changes in negative emotions less detectable. This possibility is supported by our exploratory analysis, which revealed that participants who read about the benefits of solitude reported experiencing the solitude period significantly less negatively compared to those who read the control passage. An alternative explanation echoes longitudinal work demonstrating that throughout a single day, positive emotions fluctuate three times as much as negative emotions (Trampe et al., 2015). Thus, negative emotions may be more resistant to change in such a short timeframe (i.e., 10 min). A third explanation is that our manipulations were not strong enough to produce significant changes in low‐arousal negative affect. For instance, past research indicates that single‐session mindset interventions may not be sufficiently strong to significantly improve well‐being for people with elevated mental health symptoms (Schleider & Weisz, 2018; Yeager & Dweck, 2020).

#### 5.2.3 Hypothesis 2A

Trait reappraisal—the extent to which people use reappraisal in their everyday lives to regulate their emotions—did not moderate the effect of either reappraisal condition on changes in affect. This finding diverges from Rodriguez et al. (2020), who found that trait cognitive reappraisal moderated the effects of the Loneliness De‐Biasing condition, such that individuals who used cognitive reappraisal less frequently in their daily lives benefited more from the manipulation. However, this may be due to an error in data collection (i.e., our survey recorded only 38% of trait reappraisal data) which left us underpowered to detect an effect. Alternatively, there may not have been enough variance in trait reappraisal given that lonelier people tend to be lower in trait reappraisal (Preece et al., 2021; Rodriguez et al., 2020).

#### 5.2.4 Hypothesis 2B

Perceptions of inferiority on social media significantly moderated the effect of the *Solitude Benefits* condition on changes in low‐arousal positive affect. In the *Solitude Benefits* condition, individuals who felt highly inferior to others experienced substantial increases in low‐arousal positive affect after the solitude period. However, in the *Control* condition, individuals who felt highly inferior to others experienced a decrease in low‐arousal positive affect after the solitude period. In both the *Solitude Benefits* and *Control* conditions, individuals who did not perceive themselves as inferior to others experienced similar increases in low‐arousal positive affect. This suggests that when reminded of the benefits of solitude, even people who view themselves as inferior to others can experience a boost in feelings of relaxation and contentment during solitude. Further, seeing solitude as an opportunity to improve one's well‐being may be particularly effective for those accustomed to viewing being alone as a sign of their social inadequacy.

#### 5.2.5 Hypothesis 2C

Compulsive social media use significantly moderated the effect of the *Loneliness De‐Biasing* condition on changes in low‐arousal negative affect. In the *Loneliness De‐Biasing condition,* people who do not compulsively use social media experienced an increase in low‐arousal negative affect (e.g., *bored, sad*). However, in the *Control* condition, people who do not compulsively use social media experienced a slight decrease in low‐arousal negative affect. One possible explanation is that people who less frequently use social media are not exposed to the discussion of loneliness in the media; thus, reading about the high prevalence of loneliness may paradoxically induce negative emotions for these individuals.

#### 5.2.6 Exploratory analyses

There was no effect of condition on how lonely people felt during the solitude period. Curiously, across conditions, mean levels of loneliness during the solitude period were low. This is particularly surprising given that our sample comprised individuals who were moderately to severely lonely. Why might this be? It is possible that 10 min of solitude is too short of a timeframe to substantially move the needle on loneliness. As mentioned earlier, negative emotions tend to fluctuate less throughout the day than positive emotions; thus, state loneliness may not fluctuate enough to detect meaningful changes in loneliness after 10 min of solitude (Buecker et al., 2023; Trampe et al., 2015). An alternative explanation may be that trait loneliness is not strongly related to state loneliness, as prior work has found substantial divergence between trait and state measures of various psychological phenomena (Steyer et al., 1999).

Although mean levels of loneliness during the solitude period were low, approximately 25% of people in our sample felt moderately to very lonely during the solitude period. We found that individuals who were higher in trait loneliness, who felt anxious spending time alone without their phones, and who felt uncomfortable with their thoughts felt significantly lonelier. This aligns with prior work showing that discomfort with one's thoughts is associated with loneliness (Sundqvist & Hemberg, 2021; Vanhalst et al., 2012). Specifically, people who experience intrusive thoughts or who are unable to control the content of their thoughts are more likely to feel lonely (Dorahy & Clearwater, 2012). Finally, we found that compulsive social media use and perceptions of inferiority on social media did not significantly affect how lonely participants felt during the solitude period.

### 5.3 Limitations and future directions

We acknowledge several limitations of our study design that should be addressed in future research. Here, we opted for a tightly controlled laboratory experiment to standardize the experience of solitude across participants and thereby isolate the effects of our experimental manipulations on affect. To do so, we instructed participants to sit in the same room for the same duration of time without any electronic devices. However, this task does not capture the multi‐faceted nature of solitude, so it remains unclear whether study results can be generalized to real‐life settings. In daily life, people spend time alone in diverse locations (e.g., at home, in the office, in a park) and while doing various tasks (e.g., reading, working on the computer, going for a walk; Coplan et al., 2021). Furthermore, people regularly turn to their electronic devices and social media when they are alone, which may boost their feelings of social connection and happiness (Thomas et al., 2021). Additionally, our participants spent only 10 min in solitude, and it is unknown whether cognitive change similarly improves people's emotional experiences during longer periods of solitude. Finally, participants in our study were explicitly instructed to spend time alone; thus, they did not choose to be alone. As involuntary solitude is generally less pleasant than voluntary solitude (Coplan et al., 2021; Tse et al., 2022), our study may underestimate the emotional benefits of solitude. Nonetheless, the ability to positively reframe solitude may be most important when isolation is involuntary or undesired (Rodriguez et al., 2020).

Another limitation is that although we intended to modify participants' beliefs about solitude via our reappraisal manipulations, we did not explicitly assess participants' lay beliefs about solitude. Presumably, people who already view solitude as a positive experience will not gain much from reading the *Solitude Benefits* passage. Similarly, people already aware of the “loneliness epidemic” in the United States may not benefit from reading the *Loneliness De‐Biasing* passage. People's pre‐existing beliefs about solitude—specifically, whether people perceive solitude as beneficial versus harmful to their well‐being—may moderate both the efficacy of the manipulation and the effects of solitude on emotion more generally. We encourage future work to directly assess lay beliefs about solitude and explore whether such beliefs shape people's emotional experiences of solitude in daily life.

Taken together, our study provides evidence that reframing solitude as a positive experience equips people to more readily reap its emotional benefits. Importantly, our data suggest that the emotional benefits of learning about the positive aspects of solitude are derived from cognitive change (i.e., changing how one thinks about solitude). Future research should explore the specific mechanisms that drive changes in affect during solitude. The emotional benefits of positively reframing time alone likely stem from its impact on one's thoughts and behaviors in solitude. For instance, perhaps individuals who framed time alone as a positive experience engaged in more adaptive emotion regulation strategies during the solitude period (e.g., mindfulness and self‐distancing) compared with those who did not. In the future, we plan to analyze qualitative data from our study to examine what participants were thinking about and doing during their time alone and whether these thoughts and behaviors mediated the emotional impact of reframing time alone.

Given the promise of such a brief and simple cognitive reappraisal manipulation, we encourage future research to develop more robust and intensive interventions to improve how people think about solitude. Such interventions might involve activities that are more active than reading a passage, such as having participants complete a writing task or watch an instructional video. For instance, Crum et al. (2017) found that watching a brief video clip that described the enhancing nature of stress led people to experience more adaptive emotional and cognitive responses in the face of stressors. Furthermore, although brief psychological interventions can be effective in improving well‐being in the short term, their effects are typically not sustained for more than a few weeks or months (Orosz et al., 2017; Schleider et al., 2022). Thus, future interventions that are longer in duration (e.g., that contain multiple sessions rather than a single session) may produce longer‐lasting cognitive change.

## 6 CONCLUSION

In the current study, we find that learning about the benefits of solitude leads lonely people to experience more positive emotion during a brief period of solitude. Building on past work, we demonstrate that positively reframing solitude may not only help people *conserve* positive emotion when alone—it may in fact *increase* low‐arousal positive emotion (e.g., relaxation, contentment) when alone. Critically, these findings indicate that even people who report high levels of loneliness—and are thus more likely to experience the negative effects of solitude—may benefit from shifting how they think about being alone.

## AUTHOR CONTRIBUTIONS

Micaela Rodriguez developed the study concept. Micaela Rodriguez, Benjamin W. Bellet, and Richard J. McNally designed the study. Samuel Pratt performed the data collection under the supervision of Micaela Rodriguez, Benjamin W. Bellet, and Richard J. McNally. Micaela Rodriguez conducted the data analysis and interpretation under the supervision of Benjamin W. Bellet and Richard J. McNally. Micaela Rodriguez and Samuel Pratt drafted the paper. Benjamin W. Bellet and Richard J. McNally provided critical revisions. All authors approved the final version of the manuscript for submission. Samuel Pratt and Richard J. McNally contributed to funding acquisition.

## FUNDING INFORMATION

This study was funded by a grant from the Harvard College Research Program awarded to Samuel Pratt and discretionary fundings from the Richard J. McNally Laboratory for Anxiety and Related Disorders.

## CONFLICT OF INTEREST STATEMENT

The authors declare that this study was conducted without personal, commercial, or financial relationships that could be construed as a potential conflict of interest.

## ETHICS STATEMENT

This study has been approved by the Harvard University Committee on the Use of Human Subjects (Approval No. IRB20‐0590). All participants provided informed consent before participation.

## Data Availability

The materials, datasets, and code files generated in this study can be found on a designated OSF page for this project: https://osf.io/rgcde/. The usage of these files is restricted under Creative Commons Attribution 4.0 International Public License.

## Endnotes

^1^
When the four outliers were included in the model, the ANOVA revealed no significant effect of condition on changes in LAP (Hypothesis 1A); *F*(2, 209) = 1.77, *p* = 0.17.

^2^
In a subsample of participants who met the pre‐specified threshold for loneliness at the time of the laboratory session (*n* = 151 of 212), there was a small effect of condition on changes in LAP; *F*(2, 144) = 3.43, *p* = 0.04; $\eta_{p}^{2}$ = 0.05. A post hoc analysis with the Tukey adjustment revealed that *Solitude Benefits* participants experienced a larger increase in LAP (*M* = 1.84) after the solitude period than *Control* participants (*M* = 0.34); *M*_Diff_ = 1.84, *p* = 0.03.

^3^
When the five outliers were included in the model, the ANOVA was nonsignificant for the effect on condition on change in LAN (Hypothesis 1B); *F*(2, 209) = 0.42, *p* = 0.66.

^4^
In a subsample of participants who met the pre‐specified threshold for loneliness at the time of the laboratory session (*n* = 151 of 212), there was no significant effect of condition on changes in LAN; *F*(2, 144) = 1.47, *p* = 0.23.

^5^
When the four outliers were included, the regression to detect an interaction between trait reappraisal and condition on change in LAP (Hypothesis 2A) did not yield significant cross‐products between trait reappraisal and either the *Solitude Benefits* condition, *t*(75) = 0.53, *p* = 0.60, or the *Loneliness De‐Biasing* condition, *t*(75) = 0.38, *p* = 0.70, *F*(5, 75) = 1.25, *p* = 0.29, *R* ^2^ = 0.08.

^6^
When the four outliers were included, the regression to detect an interaction between perceptions of inferiority and condition on change in LAP (Hypothesis 2B) did not yield significant cross‐products between perceptions of inferiority and either the *Solitude Benefits* condition, *t*(206) = −1.73, *p* = 0.085 or the *Loneliness De‐Biasing* condition, *t*(206) = −1.20, *p* = 0.23, *F*(5, 206) = 3.37, *p* = 0.006, *R* ^2^ = 0.08.

^7^
In a subsample of participants who met the pre‐specified threshold for loneliness at the time of the laboratory session (*n* = 151 of 212), the regression to detect an interaction between perceptions of inferiority on social media and condition on change in LAP yielded significant cross‐products between perceptions of inferiority and the *Solitude Benefits* condition, *t*(141) = −2.71, *p* = 0.008, but not the *Loneliness De‐Biasing* condition, *t*(141) = −0.28, *p* = 0.78; *F*(5, 141) = 2.76, *p* = 0.009, *R* ^2^ = 0.07.

^8^
When the four outliers were included, the regression to detect an interaction between compulsive social media use and condition on change in LAP (Hypothesis 2C) did not yield significant cross‐products between compulsive social media use and either the *Solitude Benefits* condition, *t*(206) = −0.13, *p* = 0.89, or the *Loneliness De‐Biasing* condition, *t*(206) = 1.72, *p* = 0.087; *F*(5, 206) = 1.52, *p* = 0.19, *R* ^2^ = 0.04.

^9^
When the four outliers were included, the regression to detect an interaction between compulsive social media use and condition on change in LAN (Hypothesis 2C) yielded significant cross‐products between compulsive social media use and the *Loneliness De‐Biasing* condition, *t*(206) = −1.96, *p* = 0.05, but not the *Solitude Benefits* condition, *t*(206) = 0.55, *p* = 0.58; *F*(5, 206) = 1.46, *p* = 0.20, *R* ^2^ = 0.01.

^10^
In a subsample of participants who met the pre‐specified threshold for loneliness at the time of the laboratory session (*n* = 151 of 212), the regression to detect an interaction between compulsive social media use and condition on change in LAN also did not yield significant cross‐products between compulsive social media use and either the *Solitude Benefits* condition, *t*(141) = 0.02, *p* = 0.83, or the *Loneliness De‐Biasing* condition, *t*(141) = −1.55, *p* = 0.12; *F*(5, 141) = 1.76, *p* = 0.12, *R* ^2^ = 0.03.
